# Enrichment of persisters enabled by a ß-lactam-induced filamentation method reveals their stochastic single-cell awakening

**DOI:** 10.1038/s42003-019-0672-3

**Published:** 2019-11-29

**Authors:** Etthel M. Windels, Zacchari Ben Meriem, Taiyeb Zahir, Kevin J. Verstrepen, Pascal Hersen, Bram Van den Bergh, Jan Michiels

**Affiliations:** 1 VIB Center for Microbiology, Flanders Institute for Biotechnology, Kasteelpark Arenberg 20 box 2460, 3001 Leuven, Belgium; 2 Centre of Microbial and Plant Genetics, KU Leuven, Kasteelpark Arenberg 20 box 2460, 3001 Leuven, Belgium; 3 Laboratoire Matière et Systèmes Complexes, Université Paris Diderot & CNRS UMR7057, Rue Alice Domon et Léonie Duquet 10, Paris, France

**Keywords:** Microbiology, Antibiotics

## Abstract

When exposed to lethal doses of antibiotics, bacterial populations are most often not completely eradicated. A small number of phenotypic variants, defined as ‘persisters’, are refractory to antibiotics and survive treatment. Despite their involvement in relapsing infections, processes determining phenotypic switches from and to the persister state largely remain elusive. This is mainly due to the low frequency of persisters and the lack of reliable persistence markers, both hampering studies of persistence at the single-cell level. Here we present a highly effective persister enrichment method involving cephalexin, an antibiotic that induces extensive filamentation of susceptible cells. We used our enrichment method to monitor outgrowth of *Escherichia coli* persisters at the single-cell level, thereby conclusively demonstrating that persister awakening is a stochastic phenomenon. We anticipate that our approach can have far-reaching consequences in the persistence field, by allowing single-cell studies at a much higher throughput than previously reported.

## Abstract (web summary)

Windels et al. present a method that effectively enriches persisters by separating cells tolerant to cephalexin, an antibiotic that induces filamentation of susceptible cells. This study suggests that persisters can be monitored at the single-cell level with a microfluidic device in a high-throughput manner.

## Introduction

Even in the absence of genetic resistance, antibiotic treatment often fails to completely eradicate bacterial infections. This is largely due to so-called “persister cells”, which are phenotypic variants that transiently withstand exposure to high doses of antibiotics^1,2^. Every bacterial population contains a small fraction of persisters, resulting from a phenotypic switch to an antibiotic-tolerant, dormant state. This switch is only temporary, as persisters start dividing when relieved from external stress. Persister formation and resuscitation are most often assumed to be stochastic processes directed by specific switching rates^3^, while actually very little is known about the single-cell kinetics and the underlying molecular mechanisms of these phenomena. Given the population heterogeneity involved in persistence, interrogation of the persister physiology should rely on single-cell studies to properly capture the defining traits. However, these studies require considerable and fast enrichment of persisters, as they are usually present at low frequencies and known to be in a metastable state. Problematically, apart from their antibiotic tolerance, no reliable marker currently exists to distinguish persisters from normal, susceptible cells. The state-of-the-art method to enrich for persisters involves lysis of susceptible cells by ampicillin, followed by sedimentation of intact persister cells^4^. A slightly adapted version of this method was recently also used by Pu et al.^5^. However, due to the poor separation efficiency during sedimentation, this method fails to efficiently remove cell debris and results in a persister density that is most often too low for microscopic studies. Furthermore, prolonged exposure of the culture to antibiotics or dead cell material could potentially affect persister formation^6–8^. The latter problem was addressed by Cañas-Duarte et al.^9^, who optimized a method to rapidly lyse susceptible cells using a chemo-enzymatic lysis solution. Problematically, they did not validate antibiotic tolerance of their isolated cells, nor did they report the purity and density of the resulting sample. Other approaches using GFP expression, RpoS::mCherry expression, or the RNA-binding Thioflavin T as fluorescent markers for persistence, make too strong assumptions on the physiological state of persisters and therefore generate samples that are highly contaminated with susceptible cells^10–12^. Attempts to generate persister-like cells using chemical pretreatment^8^ or strains that are engineered to accumulate toxins^13^ have yielded useful model systems, but observations in these systems always need validation in naturally occurring persisters.

In this study, we established a highly efficient persister enrichment method that largely addresses the challenges posed by single-cell persistence studies. We show that persisters can be effectively enriched by filtration after ß-lactam-induced filamentation. Cells isolated in this way are bona fide persisters as they survive during antibiotic treatment and regrow after treatment. Although not an absolute prerequisite for persistence, isolated cells also exhibit increased tolerance towards antibiotics with different targets, as many persisters have been reported to do. We then used our enrichment method to resolve a key outstanding question in the persistence field. Single-cell recovery of persisters after treatment was monitored in a microfluidic “mother machine” device. These data show that awakening of cephalexin persisters occurs at a constant rate, reflecting stochastic switching. Our approach might prove useful for future single-cell studies of persistence.

## Results

### Cephalexin treatment followed by filtration enables highly efficient enrichment of antibiotic-tolerant cells

**Fig. 1:**
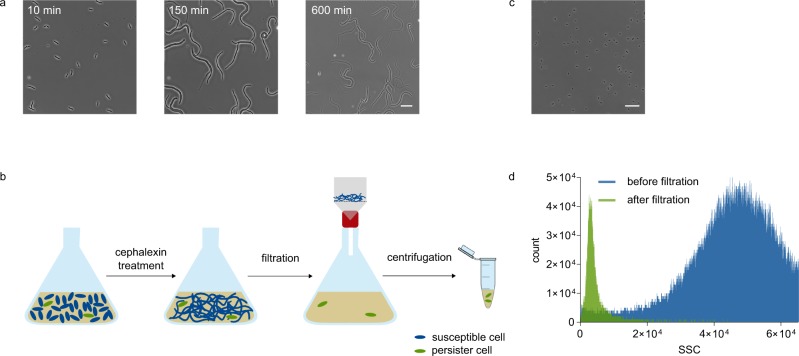
Cephalexin treatment followed by filtration enables highly efficient enrichment of antibiotic-tolerant cells. **a** Susceptible, exponential phase cells filament severely during treatment with cephalexin (50 µg/ml) before lysis occurs (scale bar: 20 µm). **b** Experimental set-up of our persister enrichment method. A culture in exponential phase is treated with cephalexin for 1 h to induce filamentation of susceptible cells. Next, the culture is vacuum filtered (pore size of 5 µm) to separate short, antibiotic-tolerant persisters from filamented, susceptible cells. After filtration, the culture is centrifuged to remove cephalexin and to increase the density of the resulting sample. **c** Microscopic visualization of a sample after cephalexin treatment and filtration demonstrates that it mainly consists of short cells that did not respond to the cephalexin treatment (scale bar: 20 µm). **d** Side scatter distributions of a sample before and after filtration confirm that filtration enriches for cells with the lowest side scatter values, presumably corresponding to the persisters of the culture (SSC: side scatter).

Similar to the ampicillin-lysis method of Keren et al.^4^, our method distinguishes persisters from susceptible cells based on their antibiotic tolerance, the core feature that universally characterizes all persisters and does not make any assumptions on their physiological state or underlying mechanisms. Our approach is specifically aimed at limiting the amount of cell debris in the resulting sample, as well as shortening the antibiotic exposure time. To this end, we benefit from the killing characteristics of cephalexin, a ß-lactam that does not immediately induce lysis, but first induces severe filamentation of susceptible cells before lysis is initiated (Fig. 1a and Supplementary Movie 1). Cephalexin targets penicillin-binding protein (PBP) 3, also known as FtsI, a transpeptidase that is essential for peptidoglycan synthesis during cell division^14^. Drug-tolerant persisters are not affected by cephalexin and therefore do not filament in its presence, enabling their enrichment from a culture by filtration (Fig. 1b). The concept of antibiotic-induced elongation followed by filtration has been introduced before^15^, but has never been optimized and validated for persister enrichment.

ß-lactams only exhibit effective activity at low-cell densities^16^, implying that cultures should be in early exponential phase when cephalexin treatment starts. The biphasic killing pattern resulting from a long-term treatment with cephalexin confirms the presence of persisters in this low-density culture (Supplementary Fig. 1a). By comparing the number of cells isolated with our filtration method to the total number of persisters calculated from a fit to the second phase of the time-kill curve (Supplementary Fig. 1a), we estimated that enrichment occurs with an average efficiency of 28%. The remaining persisters are presumably lost during filtration, as filamented cells cause clogging of the filter.

Notably, the fact that filamentation occurs at a much shorter timescale than lysis considerably reduces the antibiotic exposure time as compared to the ampicillin-lysis method. This was confirmed by performing our filtration protocol after different durations of cephalexin treatment (Supplementary Fig. 2a). These data show that the number of isolated cells does not change significantly when the preceding cephalexin treatment lasts longer than 1 h, implying that a 1-h treatment is sufficient to obtain the persisters of the culture by filtration. Any treatment shorter than 1 h results in contamination with susceptible cells, while longer treatments successively generate more debris of dead cells in the sample (Supplementary Fig. 2b). Indeed, an optimal treatment time of 1 h results in a final sample that contains short, antibiotic-tolerant cells and very little cell debris (Fig. 1c). The purity of the resulting samples was also confirmed by the side scatter distributions of samples before and after filtration (Fig. 1d).

### Cells isolated by cephalexin treatment and filtration are antibiotic-tolerant and regrow after treatment

**Fig. 2:**
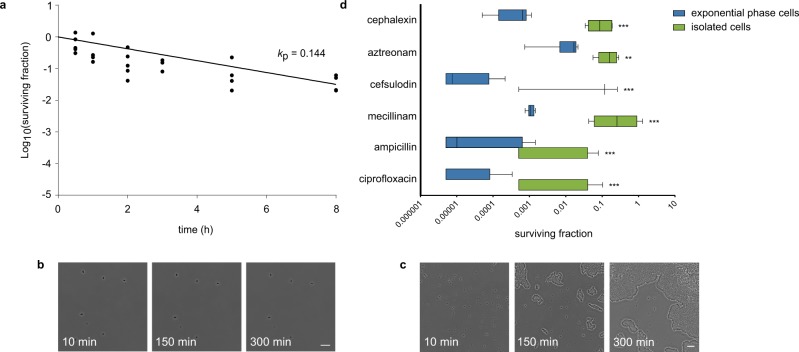
Cells isolated by cephalexin treatment and filtration are antibiotic-tolerant and regrow after treatment. **a** Time-kill curve of isolated cells treated with cephalexin (50 µg/ml) for 8 h in liquid medium (*n* = 5 biologically independent cultures). A uniphasic exponential curve was fitted onto the data with a killing rate (*k*_p_ = 0.144) that is much lower than for susceptible cells (*k*_n_ = 4.98; Supplementary Fig. 1a). The killing rate of persisters presumably reflects the rate of persister awakening in the presence of cephalexin (*k*_p_: killing rate of persisters). **b** Treatment of isolated cells with cephalexin (50 µg/ml) on an agarose pad supplemented with MHB shows that the majority of the cells is not affected by the antibiotic (scale bar: 10 µm). **c** Cells isolated by filtration start dividing on an agarose pad supplemented with MHB (scale bar: 10 µm). **d** Isolated cells display multidrug tolerance, a trait associated with persistence. Fraction of surviving cells after a 5-h treatment with cephalexin (50 µg/ml), aztreonam (0.64 µg/ml), cefsulodin (320 µg/ml), mecillinam (5 µg/ml), ampicillin (40 µg/ml), and ciprofloxacin (0.32 µg/ml), starting from an exponential phase culture or a sample consisting of isolated cephalexin persisters. For all tested antibiotics, isolated cells show a significantly higher tolerance as compared to exponential phase cells (cephalexin: *p* < 0.0001, *n* = 7 and *n* = 11 biologically independent cultures respectively; aztreonam: *p* = 0.004, *n* = 6 and *n* = 9; cefsulodin: *p* < 0.0001, *n* = 3 and *n* = 6; mecillinam: *p* < 0.0001, *n* = 5 and *n* = 9; ampicillin: *p* < 0.0001, *n* = 5 a*n*d *n* = 9; ciprofloxacin: *p* < 0.0001, *n* = 9 a*n*d *n* = 9). Whiskers represent 10–90 percentiles.

Next, we sought to validate that cells isolated by cephalexin treatment and filtration show the key properties of persisters, being the ability to survive a longer-term antibiotic treatment and to reinitiate growth after treatment. We tested the first feature by treating a sample of isolated cells with cephalexin, both in liquid medium and on an agarose pad supplemented with rich medium (Mueller-Hinton broth; MHB). In liquid culture, cephalexin causes the number of isolated cells to decline slowly (Fig. 2a), with a rate that does not significantly differ from the killing rate of persisters (*p* = 0.399; Supplementary Fig. 1). We hypothesize that this rate of killing, which is much lower than for susceptible cells, reflects the awakening rate of persisters in the presence of cephalexin^17^. Most cells on an MHB-agarose pad supplemented with cephalexin remain unaffected (Fig. 2b and Supplementary Movie 2). A few isolated cells show filamentation and lysis, which can presumably be attributed to persister awakening.

Importantly, these microscopic observations additionally demonstrate that the isolated cells cannot grow in the presence of cephalexin, implying that they are not genetically resistant. We then also validated that cells isolated by filtration are able to reinitiate growth, by seeding them onto an agarose pad supplemented with rich medium (Fig. 2c and Supplementary Movie 3). 30 to 40% of the cells started dividing within 1 h, confirming their culturability after treatment. Growth of other cells was mostly masked by colonies originating from these early-dividing cells.

Persisters are often assumed to be dormant cells in which antibiotic targets are inactive, resulting in high tolerance towards various types of antibiotics. To further confirm that cells isolated by cephalexin treatment and filtration are persisters, we investigated their tolerance towards antibiotics with cellular targets that differ from PBP3. Cefsulodin is a ß-lactam that targets PBP1a and PBP1b, while mecillinam only targets PBP2. Ampicillin has multiple targets, including PBP1a, PBP1b, PBP2, and PBP3^18^. We also investigated tolerance towards the ß-lactam aztreonam, which has the same target as cephalexin, and towards the fluoroquinolone ciprofloxacin, which targets DNA topoisomerases. Tolerance was measured by treating a sample of persisters obtained with our enrichment protocol for 5 h with the listed antibiotics. The relative fraction of surviving cells after treatment was compared to the surviving fraction of an exponential phase culture (Fig. 2d). For all antibiotics, cells surviving treatment are enriched in a culture consisting of persisters isolated by filtration (334-fold for cephalexin, 12-fold for aztreonam, 2950-fold for cefsulodin, 393-fold for mecillinam, 51-fold for ampicillin, and 368-fold for ciprofloxacin). Notably, none of the tested antibiotics results in 100% survival of the isolated persisters. In accordance with our other data (Fig. 2a), this can be partially attributed to killing of persisters as they wake up during treatment. However, these data presumably also imply that not all persisters are tolerant to all antibiotics, but rather represent a heterogeneous pool of cells with partially overlapping tolerance to various antibiotics. Altogether, our data show that cells isolated by cephalexin treatment and filtration are tolerant towards a longer-term cephalexin treatment, that they are able to reinitiate growth when treatment ceases, and that they show a high degree of multidrug tolerance. All these characteristics are key to the persister phenotype and make us confident that the culturable cells isolated by filtration are bona fide persister cells.

### Single-cell analysis of isolated persisters in the mother machine reveals that persister awakening is a stochastic process

To microscopically examine individual persister cells and their regrowth, a major drawback of using agarose pads is that early-dividing cells quickly overgrow the whole pad. First divisions of potentially later-dividing, neighboring cells are thereby obscured, hampering quantitative single-cell analyses of awakening. To address this problem, we took advantage of the mother machine, a well-established microfluidic device that allows tracking growth of a large number of individual *E. coli* cells^19^.

**Fig. 3:**
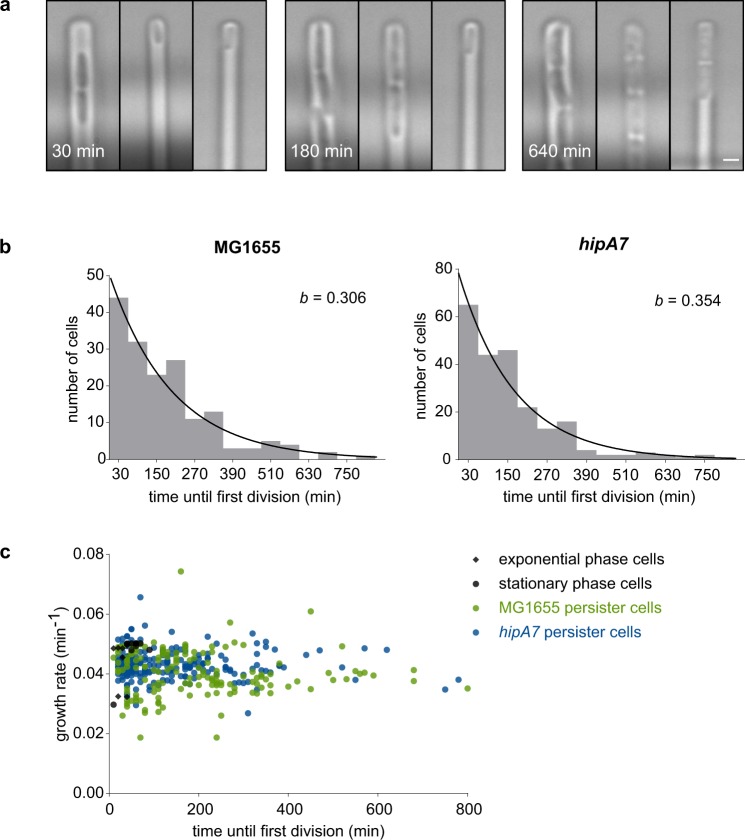
Single-cell analysis of isolated persisters in the mother machine reveals that persister awakening is a stochastic process. **a** Microscopic images of persisters dividing in the mother machine. The time until first division varies strongly among individual cells (left channel: short lag time; middle channel: medium lag time; right channel: long lag time; scale bar: 1 µm). **b** Single-cell distributions of persister awakening times measured in the mother machine, for the wild-type strain MG1655 (*n* = 174 cells) and the high-persistence strain *hipA7* (*n* = 220 cells). An exponential distribution was fitted onto the binned data, revealing an awakening rate *b* that is similar for both strains. **c** Scatterplot of single-cell awakening times and growth rates of MG1655 and *hipA7* persisters, exponential phase cells, and stationary phase cells. While awakening times show a large variation, growth rates cluster more tightly around the average value. Both parameters are not correlated.

We enriched persisters from a culture using the filtration method described above, inserted these cells into the channels of the mother machine, and provided them with fresh nutrients (Fig. 3a). Most of the channels contained either no or only one cell, which allowed to track single cells. Based on a few hundred individual observations, we derived a distribution of single-cell persister awakening times for the wild-type *E. coli* strain K-12 MG1655 and the well-known high-persistence strain *hipA7* (Fig. 3b). A similar distribution was obtained for both strains. This distribution shows a surprisingly high cell-to-cell variability in awakening times, ranging from a few minutes to up to 13 h. In both cases, an exponential curve fits well to the data, indicative of a constant rate of stochastic switching. The awakening rate in fresh medium without antibiotics (*b* = 0.31–0.35; Fig. 3b) is higher than in the presence of cephalexin (*k*_p_ = 0.04–0.18; Fig. 2a and Supplementary Fig. 1). This discrepancy might reflect a response of persisters to cephalexin, although both rates were measured in different set-ups and are therefore not perfectly comparable.

In addition to the awakening times, we also derived individual growth rates of freshly awakened persisters (Supplementary Fig. 3). Strikingly, these data reveal that persisters instantaneously divide at a rate that does not differ from the growth rate of normal cells. Furthermore, individual growth rates do not correlate with awakening times, indicating that cells with a long lag time do not necessarily grow slower than cells with a short lag time (Fig. 3c). It should be noted that the majority of the cells (60% for MG1655, 30% for *hipA7*) did not start dividing within the course of the experiment (20 h). As these cells did not stain as dead and are too numerous to be completely covered by the tail of the exponential distribution, they can presumably be classified as viable but non-culturable cells (VBNCs). The high abundance of VBNCs in *E. coli* cultures has already been reported before^20,21^ and represents a prominent source of contamination in most persistence enrichment protocols, including ours. As VBNCs cannot be distinguished from persisters based on their antibiotic tolerance, our method is only able to discriminate between both by visualizing regrowth in fresh medium.

## Discussion

Single-cell studies of persisters are usually challenging and time-consuming due to the low frequency of persisters in bacterial cultures. Here, we propose to enrich persisters by treating a culture with cephalexin, which causes filamentation of susceptible cells and thereby facilitates enrichment of antibiotic-tolerant cells by size-separation. Cephalexin exhibits activity against a wide range of bacteria, including both Gram-positive and Gram-negative bacteria, where it elicits similar effects of filamentation and lysis. Our enrichment protocol can therefore easily be extended to species other than *E. coli*. The size of the inoculum used to initiate the exponential phase culture, the duration of exponential growth, and the duration and concentration of antibiotic treatment should be optimized for every strain or species, as these parameters are highly dependent on growth rate and lag phase. Ideally, all normal cells should have escaped the lag phase, while the cell density after exponential growth should remain sufficiently low for an effective cephalexin treatment. As our method is specifically based on cell filamentation, it could also be used with other antibiotics for which this effect has been reported^22,23^.

Using our enrichment method, we found that persister awakening is a stochastic process occurring with a constant rate, which corroborates previous hypotheses. In a pioneering study, Balaban et al.^3^ proposed a quantitative model for persister formation and awakening that was conceptually based on single-cell observations of persisters originating from high-persistence mutants. However, as only a few persisters were observed microscopically, estimation of switching rates in this work was based on population-level data. In the current study we provide, to our knowledge, the first conclusive experimental evidence of stochastic awakening of wild-type persisters at single-cell level with a relatively high throughput. Furthermore, we anticipate that the wide applicability of our size-separation-based persister enrichment method will boost single-cell persistence studies, potentially with important consequences for the persistence field.

## Methods

### Strains, culture conditions, and antibiotics

Experiments were performed with *E. coli* K-12 MG1655, except when stated otherwise. MG1655 *hipA7* was constructed by Pearl et al.^24^. Strains were grown at 37 °C in Mueller-Hinton broth (MHB) with orbital shaking (200 rpm) or on Luria-Bertani (LB) agar.

### Enrichment of persisters

A 20-h overnight culture was diluted 1:10,000 in 100 ml MHB and incubated for 20 h. This culture was diluted 1:5000 in 100 ml MHB and grown for 90 min. The density of the resulting culture was 1–2 × 10^6^ CFU/ml, as determined by serial dilution in MgSO_4_ (10 mM) followed by plating on LB agar. Next, the culture was treated with cephalexin (50 µg/ml) for 60 min, after which it was poured twice over a polyvinylidene fluoride membrane filter (Merck Millipore). We used filters with a pore size of 5 µm, as normal *E. coli* cells have an average cell size of 2 µm while cephalexin-treated cells elongate to 10–100 µm. The filtrate was collected in falcons and spun down (4000 rpm—5 min). After pouring off the supernatant, the remaining volume was transferred to a microcentrifuge tube and centrifuged twice (6000 rpm—5 min) to wash away the antibiotic. The pellet was resuspended in MgSO_4_ (10 mM) or MHB.

### Time-kill curves and measurement of multidrug tolerance

A 20-h overnight culture was diluted 1:10,000 in 100 ml MHB and incubated for 20 h. This culture was then diluted 1:5000 in 100 ml MHB and grown for 90 min. The density of the resulting culture was 1–2 × 10^6^ CFU/ml, as determined by serial dilution in MgSO_4_ (10 mM) followed by plating on LB agar. To measure time-kill curves of exponential phase cells, this culture was treated with cephalexin (50 µg/ml) for 16 h. The surviving fraction was determined by serial dilution in MgSO_4_ (10 mM) and plating on LB agar, both before treatment and at specified time points during treatment. To measure multidrug tolerance of exponential phase cells, 1 ml of culture was transferred to a test tube and treated with cephalexin (50 µg/ml, 6x minimum inhibitory concentration (MIC)), aztreonam (0.64 µg/ml, 10x MIC), cefsulodin (320 µg/ml, 10x MIC), mecillinam (5 µg/ml, 40x MIC), ampicillin (40 µg/ml, 10x MIC), or ciprofloxacin (0.32 µg/ml, 20x MIC) for 5 h. The surviving fraction was determined by serial dilution in MgSO_4_ (10 mM) and plating on LB agar before and after treatment.

To measure time-kill curves of isolated cells, the filtration protocol was performed as stated above, after which the isolated cells were resuspended in fresh MHB and treated with cephalexin (50 µg/ml) for 8 h. The surviving fraction was determined by serial dilution in MgSO_4_ (10 mM) and plating on LB agar, both before treatment and at specified time points during treatment. To measure multidrug tolerance of isolated cells, isolated cells were resuspended in fresh MHB and 1 ml was treated in a test tube with cephalexin (50 µg/ml, 6x MIC), aztreonam (0.64 µg/ml, 10x MIC), cefsulodin (320 µg/ml, 10x MIC), mecillinam (5 µg/ml, 40x MIC), ampicillin (40 µg/ml, 10x MIC), or ciprofloxacin (0.32 µg/ml, 20x MIC) for 5 h. The surviving fraction was determined by serial dilution in MgSO_4_ (10 mM) and plating on LB agar before and after treatment.

### Flow cytometry

A 20-h overnight culture was diluted 1:10,000 in 100 ml MHB and incubated for 20 h. This culture was diluted 1:5000 in 100 ml MHB and grown for 90 min. Next, the culture was treated with cephalexin (50 µg/ml) for 60 min. A sample was taken from this culture and washed in (phosphate-buffered saline) PBS, after which the scattering values were measured by flow cytometry using a BD Influx cell sorter. The remainder of the culture was poured twice over a polyvinylidene fluoride membrane filter (Merck Millipore) with a pore size of 5 µm. The filtrate was collected in falcons and spun down (4000 rpm—5 min). After pouring off the supernatant, the remaining volume was transferred to a microcentrifuge tube and washed in PBS. The scattering values of this sample were measured by flow cytometry.

### Microscopy of agarose pads

To visualize killing by cephalexin, a 20-h overnight culture was diluted 1:5000 and incubated for 90 min. The resulting exponential phase culture was washed with MgSO_4_ and 2 µl of cells was seeded onto an MHB-agarose pad (2% w/v) containing cephalexin (50 µg/ml). Cells were incubated at 37 °C and killing was monitored for 6 h. Images were obtained using a Nikon Ti-E inverted microscope with a 60x objective.

To visualize persisters, cells were isolated as described above. The resulting sample was resuspended in 10 µl MgSO_4_ and 2 µl of cells was seeded onto an MHB-agarose pad (2% w/v) with or without cephalexin (50 µg/ml). Cells were incubated at 37 °C and growth was monitored for 12 h. Images were obtained using a Nikon Ti-E inverted microscope with a 60x objective.

### Fabrication of mother machine devices

Master molds of the microfluidic devices were designed and fabricated using standard microfabrication techniques^19^. Microfluidic chips were made by casting polydimethylsiloxane (PDMS) onto the wafer. PDMS (Sylgard 184 kit; Dow Corning) was prepared by mixing polymer base and curing agent in a 10:1 ratio. After degassing the mixture in a vacuum chamber, it was poured over the wafer and cured overnight at 65 °C. Devices were peeled from the wafer and holes for the inlet and outlet were punched using a syringe and needle (0.9 mm), and bonded to a glass coverslip after plasma activation. The bonding was established for at least 15 min at 65 °C. The dimensions of the growth channels were approximately 25 µm (*L*) x 1 µm (*W*) x 1 µm (*D*).

### Mother machine experiments

Persisters were enriched as described before and resuspended in a final volume of 20 µl MgSO_4_. After flushing the channels of the microfluidic device with MgSO_4_, cells were loaded by syringe injection followed by chip centrifugation. A peristaltic pump was used to flow medium through the device at a flow rate of 90 µl/min. The microscope chamber, which also contained the medium reservoir, was constantly held at 37 °C. Images were obtained using an Olympus XI71 inverted microscope with a 100x objective.

Live/dead staining was performed by flowing propidium iodide into the channels of the microfluidic device for 10 min at a concentration of 30 µM.

### Statistics and reproducibility

Images were analyzed using NIS Elements D 4.60.00 (Nikon Instruments, Japan) and ImageJ (https://imagej.nih.gov/). Flow cytometry data were analyzed with FlowJo V10. Statistical analyses were performed in R (https://www.r-project.org/).

#### Time-kill curves

Biphasic killing parameters were determined by fitting a bi-exponential mixed model to the Log_10_-transformed, normally distributed number of surviving cells (CFU/ml) using the R package *nlme* (https://cran.r-project.org/web/packages/nlme/index.html). The model was based on the equation Log_10_(CFU) = Log_10_(${N_{0}}\cdot{e^{-k_{n}\cdotτ}}+{P_{0}}\cdot{e^{-k_{p}\cdot τ}}$), with *τ* the treatment time (in hours), *N*_0_ and *P*_0_ the number of normal and persister cells at *τ* = 0, and *k*_n_ and *k*_p_ the rate at which normal and persister cells are killed (per hour)^25^. For uniphasic killing, the R package *lme4* (https://cran.r-project.org/web/packages/lme4/index.html) was used to fit the equation Log_10_(CFU) = Log_10_((*N*_0_ + *P*_0_).*e*^−*k*.*τ*^). Akaike Information Criterion was used to assess both models.

#### Multidrug tolerance

Surviving fractions were compared between conditions using one-way analysis of variance and post-hoc comparisons with Sidak’s correction for multiple testing. When no colonies were detected, the value for the surviving fraction was set at half the detection limit.

#### Distribution of awakening times

The variable “persister awakening time” was split into bins of 60 min. The number of observations in each bin was normalized, to obtain relative frequencies (freq) of awakening events. The *nls* function in R was used to fit an exponential distribution with equation Log_10_(freq) = Log_10_(*b*.*e*^−*b*.τ^) onto the data, with *τ* the time (in hours) in fresh medium and *b* the rate of awakening. After checking normality with a Shapiro–Wilk test, Log_10_-transformed awakening times were compared statistically among different strains or cell types using an unpaired, two-sided *t*-test, with Welch correction in the case of unequal variances (checked with an *F*-test).

#### Growth rates

A piecewise linear function was fitted to the cumulative number of divisions over time, for each individual cell. The number of knots was chosen by cross-validation and most often corresponds to one, dividing the growth curve into a lag phase and exponential growth phase. The slope of the second curve was then used to derive the average growth rate of individual cells. After checking normality with a Shapiro–Wilk test, growth rates were compared statistically among different strains using an unpaired, two-sided *t*-test, with Welch correction in the case of unequal variances (checked with an *F*-test).

### Reporting summary

Further information on research design is available in the Nature Research Reporting Summary linked to this article.

## Supplementary information

Supplementary Information

Description of Additional Supplementary Items

Supplementary Movie 1

Supplementary Movie 2

Supplementary Movie 3

Reporting Summary

## Data Availability

The raw data that support the findings of this study are available on FigShare 10.6084/m9.figshare.9663875^26^.
