# Supplementary material for: Enrichment of persisters enabled by a ß-lactam-induced filamentation method reveals their stochastic single-cell awakening
Source: Commun Biol. 2019 Nov 29;2:426. doi: 10.1038/s42003-019-0672-3 (PMC6884588; doi:10.1038/s42003-019-0672-3)
Supplement: Supplementary file 1 — Supplementary Information [file 42003_2019_672_MOESM1_ESM.pdf]

Supplementary Figures

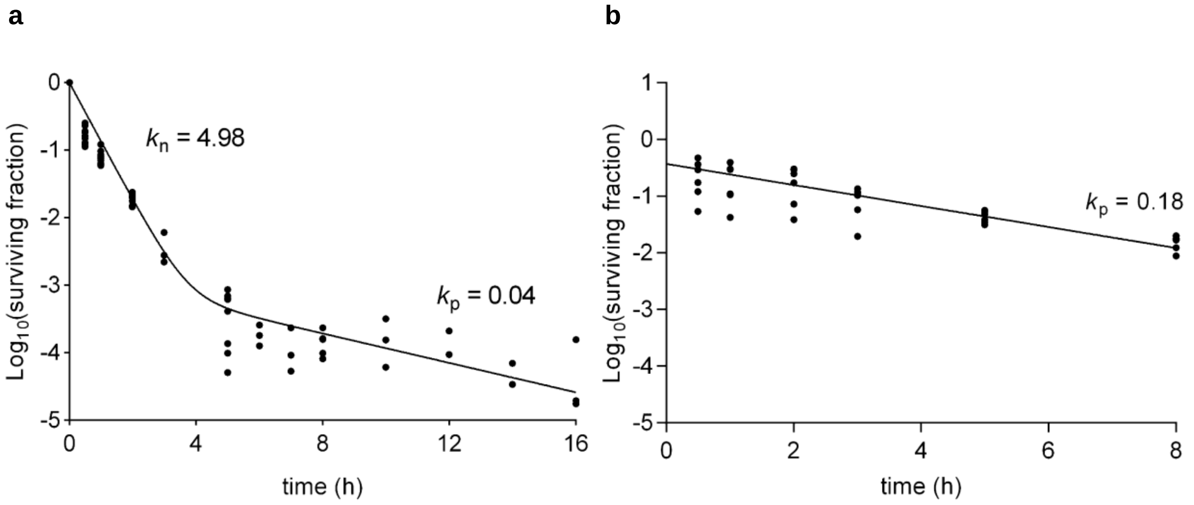

Supplementary Figure 1. Persisters are killed slowly in the presence of cephalexin. (a) Time-kill kinetics of an exponential phase culture treated with cephalexin (50 µg/ml) for 16 hours. A biphasic exponential curve was fitted onto the data, with the first phase representing the fast killing rate of susceptible cells (k_n), and the second phase showing the slow killing rate of tolerant persisters (k_p). (b) A culture was first treated with cephalexin (50 µg/ml) for 5 hours to kill all susceptible cells (not shown). The remaining persisters were then exposed to an 8-hour cephalexin treatment (50 µg/ml). A uniphasic exponential curve was fitted onto the data, with the killing rate (k_p) presumably representing the awakening rate of persisters. This rate does not significantly differ from the killing rate of cells isolated by cephalexin treatment and filtration (p=0.399; Figure 2a of the main text). Best-fit estimated values of the killing parameters are indicated on the graphs.

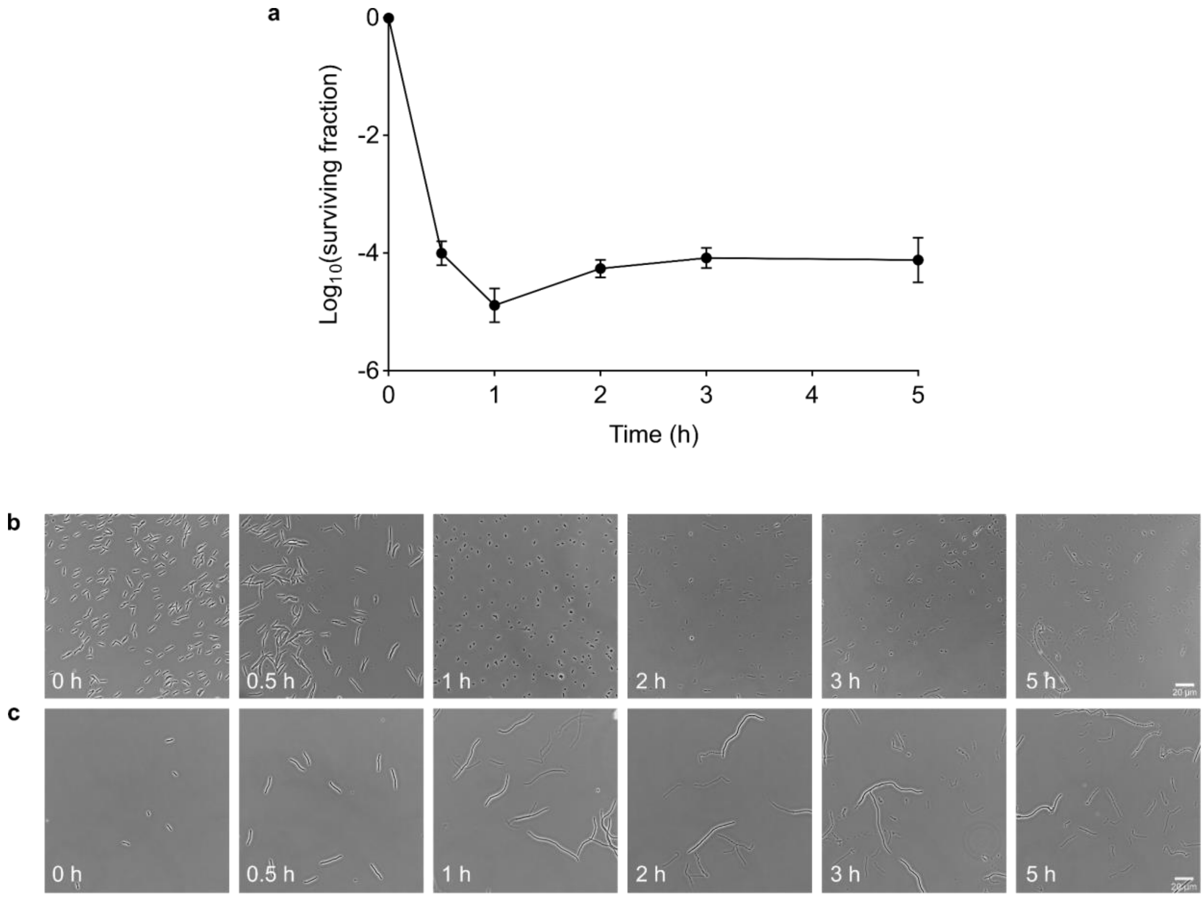

Supplementary Figure 2. A 1-hour cephalexin treatment is optimal for persister isolation by filtration. (a) Filtration was performed on samples taken at regular time points during a 5-hour cephalexin treatment (50 µg/ml). Filtration after 30 minutes of treatment results in a sample that is still contaminated with susceptible cells due to insufficient filamentation (also supported by (b)). A treatment of 1 hour is sufficient to obtain a sample only containing persisters, as the number of cells does not decrease further when the treatment is extended (*n*=3 biologically independent cultures). The latter was confirmed by fitting a linear model to the data (time ≥ 1 h). The slope of this model is not significantly different from zero (*p*=0.17). (b-c) Microscopy images of samples taken at different time points during treatment of an exponential phase culture with cephalexin (50 µg/ml), with (b) and without (c) performing filtration. A treatment that is longer than 1 hour results in an increasing amount of debris from lysed cells and might therefore hamper subsequent single-cell studies.

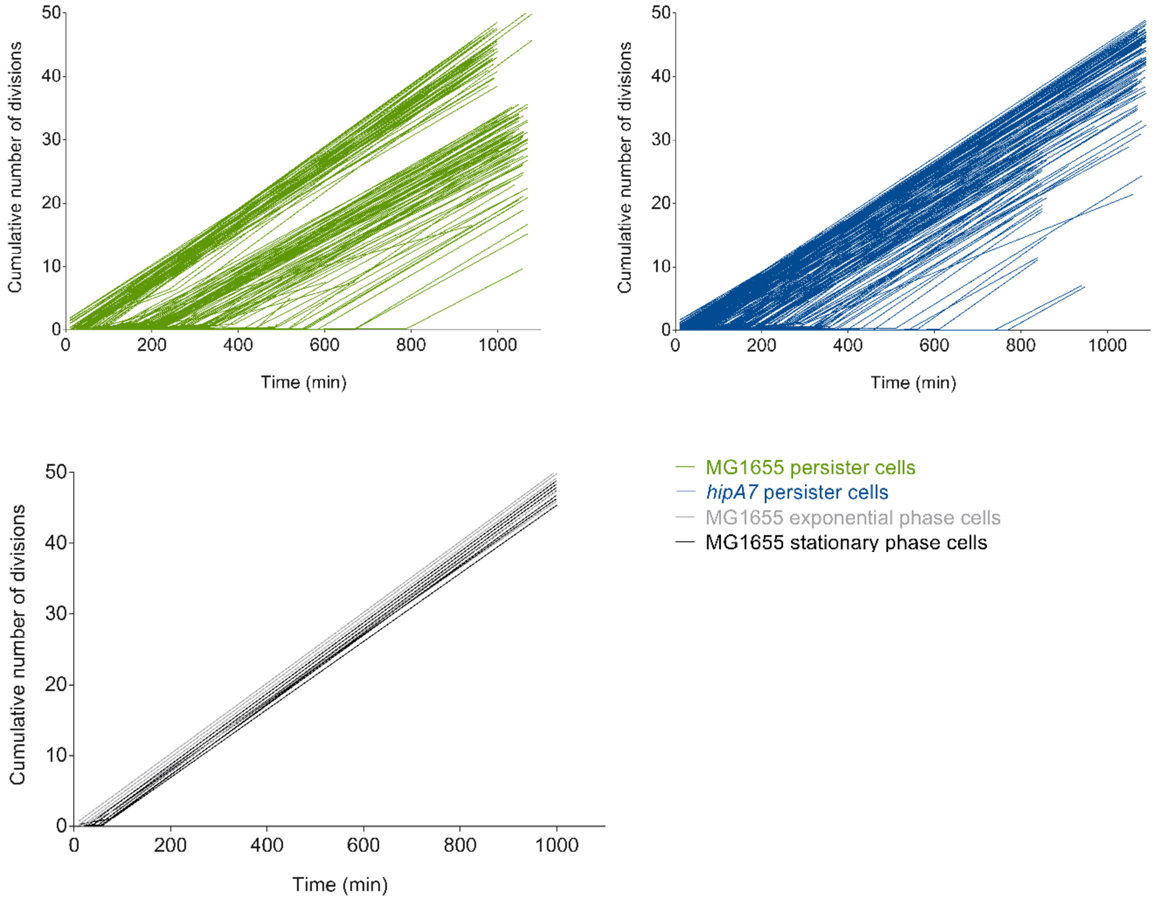

Supplementary Figure 3. Fittings to the cumulative number of divisions of awakened persisters in the mother machine. Linear splines were fitted onto the cumulative number of divisions for each awakened persister, as well as for exponential and stationary phase cells observed in the mother machine. Individual growth rates were derived from the slopes of the fitted curves (MG1655 persisters: *n*=174 cells; *hipA7* persisters: *n*=220 cells; MG1655 exponential phase cells: *n*=7 cells; MG1655 stationary phase cells: *n*=11 cells).
