# Supplementary material for: Enrichment of persisters enabled by a ß-lactam-induced filamentation method reveals their stochastic single-cell awakening
Source: Commun Biol. 2019 Nov 29;2:426. doi: 10.1038/s42003-019-0672-3 (PMC6884588; doi:10.1038/s42003-019-0672-3)
Supplement: Supplementary file 2 — Description of Additional Supplementary Items [file 42003_2019_672_MOESM2_ESM.pdf]

Description of additional supplementary items

File name: Supplementary Movie 1

Description: Susceptible, exponential phase cells on an MHB+agarose pad supplemented with cephalexin (50 µg/ml) filament severely before lysis occurs.

File name: Supplementary Movie 2

Description: Cells isolated by cephalexin treatment and filtration remain unaffected on an MHB+agarose pad supplemented with cephalexin (50 µg/ml).

File name: Supplementary Movie 3

Description: Most cells isolated by cephalexin treatment and filtration reinitiate growth on an MHB+agarose pad.
